# Long-term outcomes in elderly patients with ANCA-associated vasculitis

**DOI:** 10.1093/rheumatology/kez388

**Published:** 2019-09-17

**Authors:** Dominic McGovern, Sam P Williams, Katrina Parsons, Tariq E Farrah, Peter J Gallacher, Eve Miller-Hodges, David C Kluth, Robert W Hunter, Neeraj Dhaun

**Affiliations:** 1 Department of Renal Medicine, Royal Infirmary of Edinburgh, Edinburgh; 2 Department of Haematology, Beatson West of Scotland Cancer Centre, Glasgow; 3 University/British Heart Foundation Centre of Research Excellence, University of Edinburgh, Queen's Medical Research Institute, Edinburgh, UK

**Keywords:** ANCA, vasculitis, frailty, cyclophosphamide, rituximab, glucocorticoid, methylprednisolone

## Abstract

**Objective:**

ANCA-associated vasculitis (AAV) is a small vessel vasculitis that commonly presents in the elderly. However, there are few long-term outcome data for these patients. Here, we assessed long-term outcomes in a single-centre cohort of elderly patients with AAV. Additionally, we tested whether a pre-morbid frailty score could aid prognosis.

**Methods:**

Using a prospectively-compiled dataset, we investigated patients over the age of 65 who presented with AAV between 2005 and 2017 to a regional vasculitis centre. We used a Cox model to determine the factors associated with mortality. We also compared outcomes in pre-specified subgroups stratified by baseline frailty score, ANCA serotype and induction immunosuppression (with cyclophosphamide, rituximab or mycophenolate mofetil used as the main glucocorticoid-sparing agent).

**Results:**

83 patients were included in the study and were followed for a median of 1203 days. Median age was 74 years (range 65–92). Two- and five-year survival in the overall cohort were 83% (95% CI 75, 92%) and 75% (95% CI 65, 86%), respectively. The median cumulative dose of oral prednisolone was 2030 mg during the first three months. Only one patient received intravenous glucocorticoids. Age, frailty score and CRP at presentation were independently associated with mortality; all deaths occurred in patients aged over 75 at presentation. Patients treated with a cyclophosphamide-based induction regimen tended to be younger than those treated with rituximab or mycophenolate mofetil. Survival was better in the cyclophosphamide-treated group.

**Conclusion:**

In the contemporary era, the overall prognosis of AAV in elderly patients is good. Baseline frailty associates with disease outcomes including mortality. A low-dose glucocorticoid regimen (avoiding intravenous methylprednisolone) can be used to treat AAV effectively in elderly patients.

**Rheumatology key messages**

- A glucocorticoid-sparing regimen can give good long-term clinical outcomes in ANCA vasculitis in the elderly.
- Age, frailty score and CRP are associated with mortality in elderly patients with ANCA vasculitis.
- Patients aged >75, or with a Rockwood Clinical Frailty score of ⩾4, have higher mortality.

## Introduction

ANCA-associated vasculitis (AAV) is a small-vessel vasculitis that may present at any age. The incidence peaks in the seventh and eighth decades [1–3]. Elderly patients—often defined as being aged over 65—are particularly vulnerable to the adverse effects of the disease and of the immunosuppression used to treat it [4]. Previous studies have shown that increasing age and decreasing performance status are associated with poor outcomes. However, these studies were conducted in distinct patient sub-groups, such as those with ANCA-associated glomerulonephritis, and in an era pre-dating the widespread use of rituximab [5–8]. Also, elderly patients are under-represented in randomized controlled trials of induction immunosuppression in AAV. For example, mean participant ages in the CYCLOPS, RAVE and MYCYC trials were 57, 53 and 60 years, respectively [9–11].

It is important to accurately define the outcomes of AAV in the elderly so that doctors, patients and their families are able to make informed choices about treatment. We therefore aimed to assess long-term outcomes in an unselected ‘real-world’ cohort of patients with AAV presenting at the age of 65 years and over. We tested the hypothesis that a baseline frailty score would provide prognostic information.

## Methods

### Patient population and data collection

We recruited consecutive patients with a diagnosis of AAV who presented to our vasculitis service at age 65 or over from 1 July 2005 to 9 November 2017. The Royal Infirmary of Edinburgh provides a vasculitis service for a population of around one million. We have previously described the structure of our clinical service [12]. The majority of new patients are referred from secondary care; around 20% are referred from general practice or accident & emergency. In addition to referrals made on the basis of a clinical suspicion of ANCA vasculitis, all new positive ANCA results in Lothian are reflexed to a senior vasculitis clinician who then discusses the result with the physician who requested the test. Clinical and laboratory data were collected prospectively. eGFR was calculated using the CKD-EPI equation. Vasculitis disease activity was assessed at each clinic visit. We recorded the cumulative dose of glucocorticoids administered during the first 3 months. The following adverse events were recorded: infections requiring hospitalization and/or intravenous antibiotic therapy, fractures, new diagnosis of malignancy, cardiovascular/venous thromboembolic events and death. As this analysis formed part of a clinical service evaluation, we did not obtain specific ethical approval or individual patient consent.

### Frailty score

Baseline frailty was scored using a validated clinical scale: the nine-point Canadian Study on Health and Ageing Clinical Frailty Scale, also known as Rockwood’s Clinical Frailty Scale (RCFS). This tool has been shown to have good inter-observer variability and associates with hard outcomes in a range of diseases [13–18]. As well as evaluating the potential utility of the RCFS on the nine-point scale, we also conducted a pre-specified analysis comparing outcomes in patients deemed to be ‘less frail’ or ‘more frail’ at baseline (RCFS ⩽3 *vs* ⩾4). This RCFS threshold was chosen as it is the point at which symptoms begin to limit activities and is thus a relatively sensitive measure of vulnerability and frailty. A score of 3 is given to patients who are, ‘Managing Well – People whose medical problems are well controlled, but are not regularly active beyond routine walking’. A score of 4 is for those who are, ‘Vulnerable – While not dependent on others for daily help, often symptoms limit activities. A common complaint is being “slowed up,” and/or being tired during the day’.

### Disease and treatment definitions

Remission was defined as clinically silent disease in a patient taking no more than 7.5 mg prednisolone per day [19]. In this context, clinically silent disease was defined as the absence of symptoms or signs attributable to vasculitis disease activity. Relapse was defined as a change in symptoms or signs that—after appropriate investigation—was likely to be attributable to vasculitis disease activity and not to alternative pathology such as infection. In almost all instances this prompted an escalation in immunosuppression. Induction regimens were classified as ‘cyclophosphamide-based’ if the patient received cyclophosphamide exclusively, ‘rituximab-based’ if the patient received rituximab exclusively or in conjunction with up to two doses of intravenous cyclophosphamide, and ‘MMF-based’ if patients received mycophenolate mofetil (MMF) but neither cyclophosphamide nor rituximab (with or without glucocorticoids).

Oral prednisolone was administered according to a regimen tailored to the individual patient. For most patients, this was initiated at a daily dose of 1 mg per kg body weight (usually no more than 60 mg). The dose was then reduced according to the regimen outlined in Supplementary Table S1, available at *Rheumatology* online. This was tailored according to patient and disease characteristics (age, frailty, co-morbidities, infections, severity of vasculitis etc.). We do not routinely prescribe intravenous glucocorticoids at presentation (for patients of any age).

### Statistical analysis

Statistical analysis was conducted in R (version 3.5.3) [20]. Unless stated otherwise, data are presented as median and interquartile range. Non-parametric data were compared by the Wilcoxon rank sum test. Survival analysis was performed using the Survminer package [21]. Mortality risk was compared between groups by determining Cox proportional hazard ratios with associated 95% CIs, after first evaluating Schoenfield residuals to confirm that the proportional hazards assumption was satisfied. We adjusted for frailty, age, sex, ANCA status, eGFR and CRP, all assessed at baseline (i.e. time of patient presentation). In the adjusted model, eGFR was treated as a continuous variable as it demonstrated linearity. For eGFR, the hazard was calculated in decile (10 ml/min) rather than 1 ml/min increments to better reflect clinically meaningful incremental changes in renal function. CRP did not demonstrate linearity and was therefore analysed as a categorical variable using thresholds of 25, 50, 100 and 150 mg/l, chosen to correspond to clinically meaningful increments in the inflammatory response.

## Results

### Baseline demographic and disease characteristics

Eighty-three patients were included and were followed up for a median of 1203 days (interquartile range (IQR) 664–2166). Baseline data are presented in Table 1. The median age was 74 years (Supplementary Fig. S1A, available at *Rheumatology* online); 63% of patients were female. Fifty-seven patients were positive for anti-MPO ANCA, 22 for anti-proteinase 3 (PR3) ANCA, two had both and two were ANCA-negative. The distribution of frailty scores is shown in Supplementary Fig. S1B, available at *Rheumatology* online.

**Table 1:** Baseline characteristics of the study cohort

ANCA serotype	MPO (*n* = 57)	PR3 (*n* = 22)	Other (*n* = 4)	Overall (*n* = 83)
**Demographic data**				
Age (years)	73 (68, 80)	74 (66, 78)	82 (77, 84)	74 (68, 80)
Female	36 (63%)	13 (59%)	3 (75%)	52 (63%)
Weight (kg)	72 (60, 85)	74 (67, 83)	62 (61, 62)	72 (60, 84)
RCFS	3.00	3.00	3.00	3.00
	(2.75, 4.00)	(3.00, 4.00)	(2.75, 3.75)	(3.00, 4.00)
**Laboratory data**				
Creatinine (microM)	233 (142, 345)	215 (89, 357)	130 (63, 212)	221 (130, 338)
eGFR (ml/min)	38 (22, 70)	40 (17, 90)	74 (40, 105)	40 (22, 77)
U.PCR (mg/mmol)	109 (47, 274)	211 (33, 283)	47 (36, 310)	121 (45, 285)
Haemoglobin (g/L)	96 (83, 106)	94 (84, 107)	112 (94, 129)	96 (84, 107)
Albumin (g/L)	27 (21, 33)	26 (21, 31)	28 (24, 32)	27 (21, 33)
CRP (mg/L)	56 (14, 112)	70 (26, 144)	38 (22, 58)	58 (15, 112)
**Organ involvement**				
Kidney	53 (91%)	18 (86%)	2 (50%)	73 (83%)
Lung	27 (47%)	13 (62%)	2 (50%)	42 (51%)
Nerve	17 (29%)	4 (19%)	2 (50%)	23 (28%)
ENT	9 (16%)	4 (19%)	1 (25%)	13 (16%)
Skin	6 (10%)	6 (29%)	0 (0%)	12 (14%)
Joints	3 (5%)	4 (19%)	0 (0%)	7 (8%)
Eyes	2 (3%)	3 (14%)	0 (0%)	5 (6%)
Pulmonary haemorrhage	2 (3%)	2 (10%)	1 (25%)	5 (6%)
**Induction regimen**				
Glucocorticoids	57 (98%)	20 (95%)	3 (75%)	80 (96%)
Cyclophosphamide	22 (38%)	10 (48%)	0 (0%)	32 (39%)
Rituximab	24 (41%)	6 (29%)	1 (25%)	31 (37%)
MMF	10 (17%)	0 (0%)	0 (0%)	10 (12%)
Other	0 (0%)	4 (19%)	2 (50%)	6 (7%)
Plasmapheresis	15 (26%)	7 (33%)	0 (0%)	22 (27%)

Data are median (IQR) unless stated otherwise. RCFS, Rockwood Clinical Frailty Scale; eGFR, estimated glomerular filtration rate; U.PCR, urine protein : creatinine ratio.

Fifty-seven patients (68%) had involvement of more than one organ at diagnosis. Around 50% of patients had respiratory system involvement at presentation but only five patients (6% of the whole cohort) had alveolar haemorrhage.

### Treatment

Eighty patients (96%) were treated with oral prednisolone. The median cumulative dose of prednisolone administered during the first 3 months was 2030 mg (IQR 1785–2167). There was no significant difference in the cumulative glucocorticoid exposure in the more and less frail groups (Supplementary Fig. S1C, available at *Rheumatology* online). Only one patient was prescribed intravenous methylprednisolone. Three patients were treated with an entirely glucocorticoid-free regimen.

Thirty-two (39%) patients were treated with a cyclophosphamide-based induction regime, 31 (37%) with a rituximab-based regime, 10 (12%) with MMF and 10 (12%) with alternative glucocorticoid-sparing agents (e.g. methotrexate or no steroid-sparing agent). Twenty-two patients received adjunctive plasmapheresis; these patients all had aggressive disease (as assessed clinically and from the kidney biopsy) or alveolar haemorrhage. We have previously described our criteria for administering plasmapheresis (in the pre-PEXIVAS era) [12]. All patients received co-trimoxazole for *Pneumocystis jirovecii* prophylaxis. Median duration of the presenting hospital admission was 16 days (IQR 8–27 days).

### Outcomes

**Fig. 1:**
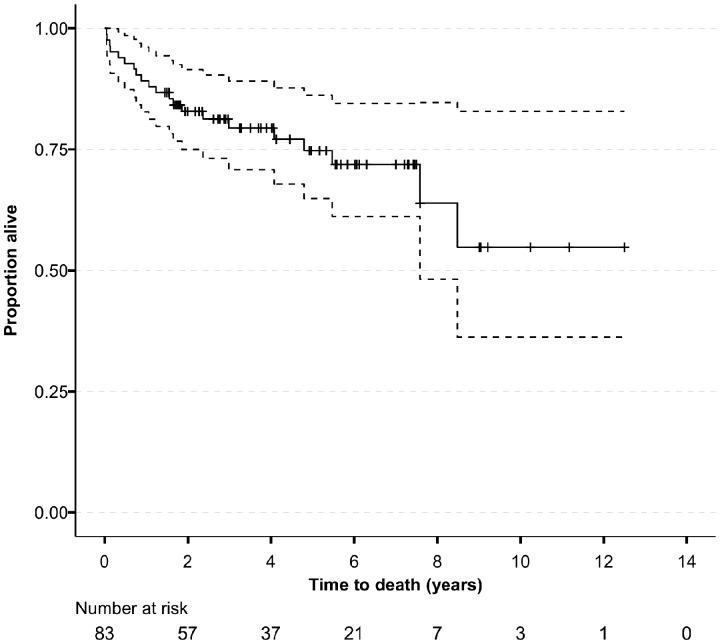
Patient survival in whole cohort (with 95% CI) .

During follow-up, 21 patients (25%) died; median time to death was 453 days (IQR 176–1090). Two-year and five-year survival in the whole cohort were 83% (95% CI 75, 92%) and 75% (95% CI 65, 86%), respectively (Fig. 1). Remission was achieved in 70 patients after a median of 125 days (IQR 92–179). Disease relapsed in 16 of these patients after a median of 632 days (IQR 326–915) from the date of remission. Five patients had more than one disease relapse.

### Adverse events

Seventy-four adverse events were recorded. The most common adverse event was infection requiring hospitalization (44 events). Other adverse events were venous thromboembolism (9), skeletal fracture (8), acute coronary syndrome (6) and new malignancy (6).

### Factors associated with mortality

**Fig. 2:**
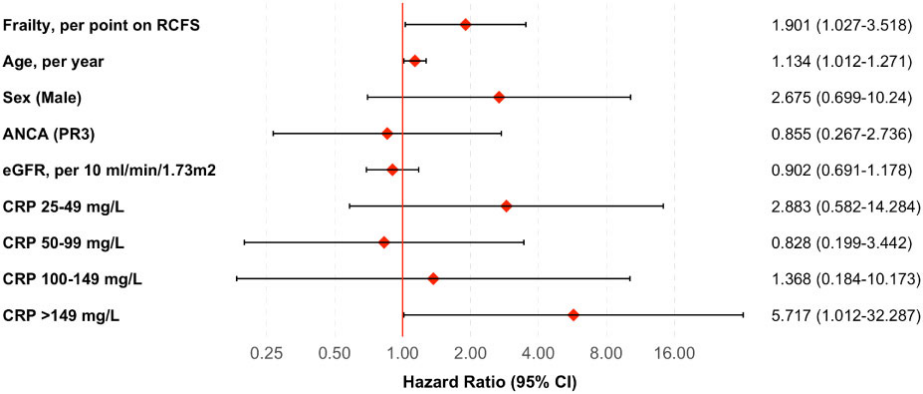
Hazard of death during follow-up period Hazard ratios (with 95% CI) were computed in a Cox proportional hazards model, adjusted for frailty, age, sex, ANCA status, eGFR and CRP. All predictor variables were assessed at presentation. RCFS, Rockwood Clinical Frailty Scale; eGFR, estimate glomerular filtration rate.

In order to identify the factors that are associated with mortality, we constructed a Cox proportional hazards model. Frailty score, age and very high baseline CRP (> 149 mg/l) were independently associated with increased risk of death (Fig. 2). Indeed, all of the deaths occurred in patients aged over 75.

### Associations with ANCA serotype

**Fig. 3:**
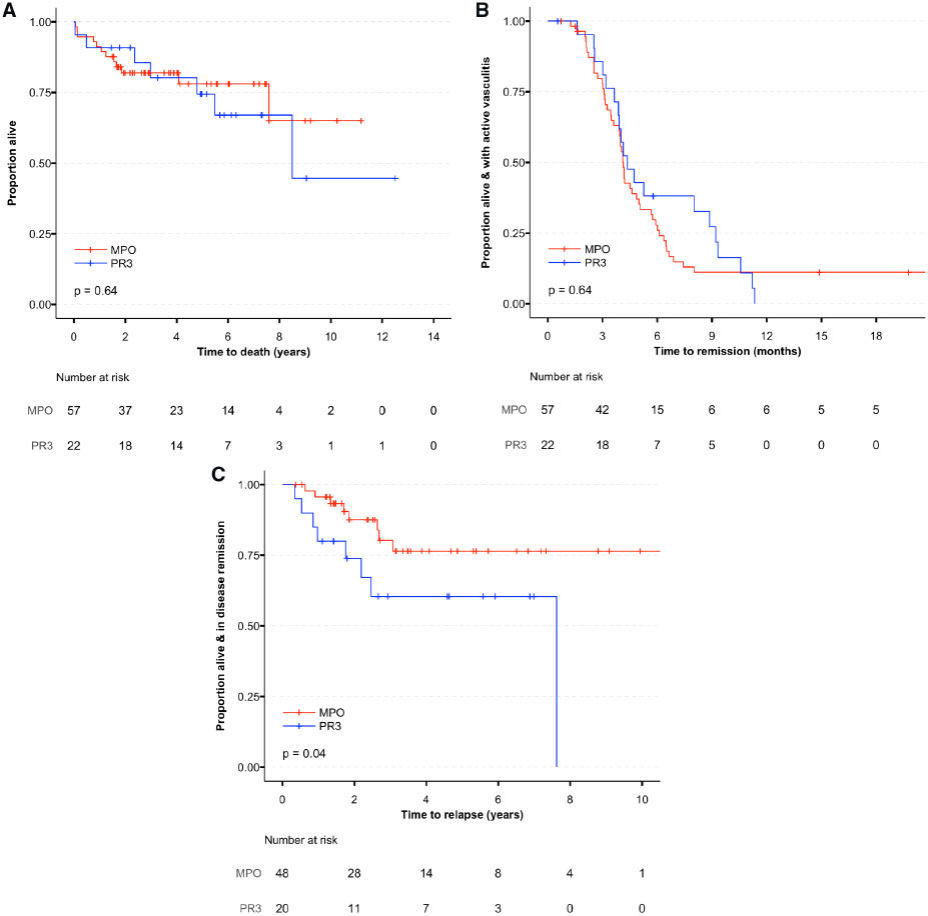
Disease outcomes stratified by ANCA serotype **A)** Time to death. **B)** Time to remission. **C)** Time to relapse.

As expected, there were differences between MPO- and PR3-positive patients at baseline, with PR3-positive patients being more likely to have lung, skin, joint and eye involvement (Table 1). PR3-positive patients were also more likely to have alveolar haemorrhage (10% *vs* 4%). There were no differences in mortality or time-to-remission between PR3- and MPO-positive patients but PR3-positive patients had a higher rate of relapse and relapsed earlier (Fig. 3).

### Associations with baseline frailty score

Baseline frailty was associated with mortality. The unadjusted hazard ratio was 2.07 (95% CI 1.50, 2.87), meaning that for each additional point on the frailty score, the risk of death approximately doubled. In a model adjusted for potentially confounding variables (age, sex, ANCA status, eGFR and CRP), the hazard ratio was 1.90 (1.03–3.52) (Fig. 2).

In a pre-specified analysis, we compared outcomes in those patients with a lower *vs* higher baseline frailty score (RCFS ⩽3 *vs* ⩾4). (This is the point at which frailty begins to limit activity, see Methods.) Fifty-five patients were in the less frail group; 27 were in frailer group; the remaining patient had no baseline frailty score recorded. Mortality was significantly higher in the frailer group. Two-year survival was 63% *vs* 93%; five-year survival 47% *vs* 90%. There were no differences between groups in time to remission or time to relapse (data not shown). There were more adverse events in the frailer group (1.4 *vs* 1.1 events per patient) and a greater proportion of patients in the frailer group had at least one adverse event (81% *vs* 58%; *P =* 0.065 by χ^2^ test). Patients in the frailer group had a longer index hospital admission (23.7 ± 16.4 *vs* 15.0 ± 13.0 days; *P* = 0.02).

### Associations with induction immunosuppression regimen

The cohorts treated with cyclophosphamide, rituximab and MMF differed in their baseline characteristics (Table 2 and Supplementary Fig. S2, available at *Rheumatology* online). Patients treated with cyclophosphamide tended to be younger and have better renal function at presentation. Mortality was marginally lower in the cyclophosphamide group (Supplementary Fig. S3, available at *Rheumatology* online); there were no differences between treatment groups in time to remission or time to relapse (data not shown). The proportion of patients who experienced at least one adverse event did not differ between groups (rituximab 61%, cyclophosphamide 75%, MMF 70%; *P =* 0.501 by χ^2^ test).

**Table 2:** Baseline characteristics, stratified by induction regimen

Induction regimen	RTX (*n* = 31)	CYC (*n* = 32)	MMF (*n* = 10)	other (*n* = 10)
**Demographic data**				
Age	79 (74, 82)	68 (66, 72)	78 (74, 83)	82 (73, 86)
Female (%)	14 (45)	21 (66)	8 (80)	9 (90)
Weight (kg)	73 (59, 83)	74 (66, 80)	67 (58, 86)	63 (56, 76)
MPO (%)	22 (71)	22 (69)	10 (100)	3 (30)
PR3 (%)	8 (26)	10 (31)	0 (0)	4 (40)
RCFS	3 (3.00, 4.00)	3 (2.00, 3.00)	3.50 (3.00, 5.00)	3.50 (3.00, 5.75)
**laboratory data**				
Cr (microM)	264 (201, 360)	179 (86, 266)	253 (156, 336)	153 (66, 208)
eGFR (ml/min)	25 (18, 41)	47 (27, 96)	32 (24, 61)	68 (34, 104)
U.PCR (mg/mmol)	121 (50, 212)	112 (44, 284)	290 (154, 427)	260 (41, 497)
Hb (g/L)	98 (92, 105)	94 (82, 110)	92 (79, 106)	85 (84, 120)
alb (g/L)	26 (21, 32)	25 (20, 32)	29 (27, 36)	30 (25, 35)
CRP (mg/L)	61 (24, 104)	70 (14, 158)	38 (20, 62)	55 (15, 87)

Data are median (IQR) unless stated otherwise. RCFS, Rockwood Clinical Frailty Scale; eGFR, estimated glomerular filtration rate; U.PCR, urine protein : creatinine ratio; Hb, haemoglobin.

## Discussion

We present the long-term outcomes of an unselected cohort of elderly patients with AAV. Mortality and morbidity outcomes were favourable, supporting the view that there is overall benefit in treating elderly patients presenting with AAV. Our cohort is representative of other studies in elderly patients with AAV, with a relatively high proportion of MPO-positive disease [6, 22–24]. However, our study cohort is larger and has longer follow-up than most published observational and interventional studies of AAV in the elderly.

In our cohort (median age 74), two-year and five-year survival were 83% and 75%, respectively. To put this into context, the two-year survival of a Scottish 74-year-old is ∼94% in the general population [25]. For Scottish adults with type 1 diabetes mellitus, two-year survival in this age-group is ∼86% [26].

### Associations with mortality

It is not surprising that increasing age was independently associated with mortality. That a frailty score was also associated with mortality—even after adjusting for age and other potentially confounding variables—suggests that this simple tool might provide useful additional information about elderly patients with ANCA vasculitis. Baseline frailty was associated with greater mortality, more adverse events and a longer hospital stay. This, therefore, has the potential to provide a free, accessible and rapid tool to allow prognostication in elderly patients presenting with AAV. Such information is invaluable to clinicians, patients and their families when deciding whether and how to treat their disease. Our data emphasize that advanced care planning should be a core component of care for frail, elderly patients presenting with AAV, and particularly in patients aged over 75. Our data could inform the design of future interventional randomized controlled trials by defining a cohort of frail elderly patients who might benefit from more conservative (e.g. glucocorticoid-free) treatment regimens.

### Induction immunosuppression

In our study, the choice of induction regimen was not protocolised. This was selected at the discretion of a senior vasculitis physician. We tended to use cyclophosphamide in patients who were younger and who had relatively better renal function. The better survival in the cyclophosphamide group is therefore likely to reflect baseline patient characteristics rather than any effect of the treatment itself (i.e. confounding by indication). Nevertheless, our data demonstrate that rituximab and MMF are useful options for treating the frail elderly in a ‘real-world’ setting. As in randomized controlled trials, rituximab was non-inferior to cyclophosphamide at inducing and maintaining disease remission and in its adverse event profile [10].

We used a relatively low-dose glucocorticoid regime. Our median cumulative dose of oral prednisolone was ∼2 g during the first three months. This equates to an average daily dose of ∼22 mg. Following our typical regimen (Supplementary Table S1, available at *Rheumatology* online), we would be predicted to prescribe a cumulative dose of ∼2.1 g during the first three months. To put this in context, the ‘predicted’ cumulative dose is ∼1.8 g in the low-dose glucocorticoid arm of the PEXIVAS trial and ∼3.3 g in the standard-dose arm [27]. (Data on the actual doses administered in PEXIVAS are not yet in the public domain.) A recent study reported outcomes in 49 patients treated with a glucocorticoid-sparing regime. Here, the investigators used a cumulative dose of <0.7 g within the first three months [28]. However, and in contrast to our cohort, patients in this study and in PEXIVAS were routinely treated with intravenous methylprednisolone (up to 3 g) at disease presentation. Therefore, our glucocorticoid regimen is low-dose compared with other published observational and interventional studies. Our favourable outcomes add to the growing weight of evidence that glucocorticoid doses can be safely minimized in AAV. They also suggest that— particularly in elderly patients— intravenous methylprednisolone is not a necessary component of induction immunosuppression for the vast majority of patients. Our findings therefore align with those of a recent retrospective analysis of short-term outcomes in severe renal AAV. In this group, IV methylprednisolone use was associated with increased risks of infection and diabetes (and not with any differences in patient or renal survival) [29].

### Strengths and limitations

The observational nature of our study has strengths and limitations. We cannot determine the effect of any treatment regimen on clinical outcome. However, as the results of randomized controlled trials are not always borne out in ‘real-world’ clinical practice, our data provide valuable information on disease outcomes in a contemporary patient cohort.

Similarly, given the single-centre nature of our study, our findings cannot be extrapolated widely without replication. Conversely, as all immunosuppression was prescribed by two senior clinicians, our data are not subject to the noise generated by inter-individual and inter-centre variability, allowing us to resolve those patient and disease factors that influence prescribing. Moreover, the robust systems used in our centre for prospective data collection mean that we have a near-complete dataset.

Published trials in ANCA vasculitis tend to report outcomes in tertiary referral centres with a specialized vasculitis service. This may of course bias the literature towards more favourable outcomes (and any *publication* bias might be expected to contribute further to this distortion). Our study— performed in one such specialized vasculitis service—should be interpreted with these caveats in mind. To our knowledge, the hypothesis that a specialized vasculitis service results in better patient outcomes has not been formally tested; this is a potentially important topic for future research.

Other potential sources of bias in this observational study are case ascertainment and referral bias: i.e. it is possible that elderly and/or frail patients are less likely to be investigated for (and therefore diagnosed with) ANCA vasculitis. The apparent decline in the incidence of ANCA vasculitis in patients aged over 80 suggests that our data may be subject to such a bias (Supplementary Fig. S1, available at *Rheumatology* online).

### Conclusions

We have presented the long-term outcomes in an unselected cohort of elderly patients with AAV. Overall, two-year and five-year survival rates were 83 and 75%, respectively. Increasing age, frailty and very high CRP at baseline were associated with mortality. A higher baseline frailty score was associated with increased mortality and more adverse events. Cyclophosphamide, rituximab and MMF are all valid treatment options in the elderly. A low-dose glucocorticoid regimen (in which intravenous methylprednisolone is not routinely used) can be used to treat AAV in elderly patients, with favourable outcomes.

## Supplementary Material

kez388_Supplementary_Data.docx
