# Supplementary material for: Long-term outcomes in elderly patients with ANCA-associated vasculitis
Source: Rheumatology (Oxford). 2019 Sep 17;59(5):1076–83. doi: 10.1093/rheumatology/kez388 (PMC7671635; doi:10.1093/rheumatology/kez388)
Supplement: kez388_Supplementary_Data.docx [file kez388_supplementary_data.docx]

# **SUPPLEMENTARY MATERIAL**

**Supplementary Table S1 – Standard glucocorticoid regimen**

| **Time-point (relative to presentation)** | **Daily dose of oral prednisolone (mg)** |
| --- | --- |
| week 1 | 1 mg per kg body weight (usually no more than 60 mg) |
| week 2 | 45 |
| week 3 | 30 |
| week 4 | 25 |
| week 5 | 20 |
| week 7 | 17.5 |
| week 9 | 15.0 |
| week 11 | 12.5 |
| week 13 | 10.0 |
| month 6 | 7.5 |
| month 9 | 5.0 |
| month 12 – 18 | stop |

Patients were not routinely treated with intravenous methylprednisolone. Prednisolone dose was tailored for each patient as described in the main text.

**Supplementary figure legends**

**Supplementary Figure S1 – Age and frailty in the study cohort. A)** Distribution of age at presentation. **B)** Distribution of frailty score at presentation. **C)** Cumulative dose of oral prednisolone administered during the first three months. The cumulative dose did not differ significantly between the frailer (RCFS >= 4) and less frail (RCFS <= 3) groups (p = 0.35 by Wilcoxon rank sum test).

**Supplementary Figure S2 – Baseline characteristics, stratified by induction regimen. A)** Age. **B)** Frailty score (RCFS, Rockwood’s Clinical Frailty Score).

**Supplementary Figure S3 – Mortality, stratified by induction regimen.**
